# Prolonged Survival in a Patient With Extensive-Stage Small Cell Lung Cancer in Spite of Discontinued Immunotherapy With Atezolizumab

**DOI:** 10.7759/cureus.37757

**Published:** 2023-04-18

**Authors:** Kazuhisa Konishi, Hiroomi Kuwahara, Daiki Morita, Shunsuke Imai, Kazuhiro Nagata

**Affiliations:** 1 Department of Respiratory Medicine, Koseikai Takeda Hospital, Kyoto, JPN; 2 Department of Pathology, Koseikai Takeda Hospital, Kyoto, JPN

**Keywords:** small cell lung cancer (sclc), cancer survival, atezolizumab, immune-checkpoint inhibitors, extended stage small cell lung cancer (es-sclc)

## Abstract

A 64-year-old man was referred from a local clinic with a chief complaint of cough. Computed tomography (CT) revealed a mass comprising a tumor in the right lower lobe and enlarged mediastinal lymph nodes, and a whole-body workup with positron emission tomography-CT showed bilateral lymph node enlargement and cancerous pericarditis. Biopsy with bronchoscopy of the right lower lobe tumor and mediastinal lymph node confirmed the histological findings of small cell lung carcinoma. The clinical diagnosis of extensive-stage small cell lung cancer (ES-SCLC) was confirmed, and first-line treatment with carboplatin, etoposide, and atezolizumab was initiated, followed by tri-weekly atezolizumab thrice. The patient experienced worsening pleural effusion treated with thoracentesis, pleural drainage, and pleurodesis. He also experienced several recurrences, which were managed with second and third-line chemotherapy with nogitecan and amrubicin. He has been receiving third-line therapy for over 30 months since his initial visit and remains stable as of today. The patient experienced an exceptional treatment outcome considering that the prognosis of ES-SCLC remains poor, with a median survival of approximately 10 months with conventional chemotherapies using cytotoxic agents. The use of immune checkpoint inhibitors (ICI) for ES-SCLC as first-line treatment may demonstrate a persistent antitumor effect, and result in improved survival following discontinuation. In conclusion, therapy including ICI for patients with ES-SCLC is a treatment option that shows possibilities in improving survival even after discontinuation.

## Introduction

Most patients diagnosed with extended-stage small cell lung cancer (ES-SCLC) have a poor prognosis, with conventional chemotherapies using cytotoxic agents offering a median survival of approximately 10 months [1]. With the recent introduction of immune checkpoint inhibitors (ICI), some lung cancer patients experience outstanding therapeutic results, indicating improved antitumor effects and longer survival [2]. ICI are monoclonal antibodies and exert its pharmacological effect by binding to PD-L1 protein. ICI allow the immune system to demonstrate an enhanced antitumor effect by blocking the immune mechanism called PD-L1 checkpoint, which inhibits the immune system's ability to recognize and degrade malignant cells [3]. In addition to its direct effects on the immune system, ICI may also have other indirect effects on the tumor microenvironment, including its ability to recruit other immune cells to the tumor site, or it may reduce the production of factors that promote tumor growth and survival [4]. Some studies have also reported that a smaller proportion of patients treated with ICI experience long-term disease control and survival, with some patients remaining disease-free for several years [5]. While ICI treatments demonstrate possibilities to improve primary outcomes, predicting which patients will benefit most from these treatments remains an active area of research [6]. Additionally, the consequences of discontinued ICI treatment in ES-SCLC patients following recurrences are still not well evaluated, and conventional therapies with cytotoxic agents beyond second-line therapies offered limited treatment outcomes [7]. Therefore it remains a challenge for the majority of ES-SCLC patients experiencing recurrences to obtain a prognosis for years, and the efficacy of ICI in ES-SCLC is an area of ongoing studies. Here we report a case of ES-SCLC with prolonged survival of over 30 months, despite multiple recurrences following discontinued treatment with atezolizumab.

## Case presentation

**Figure 1:**
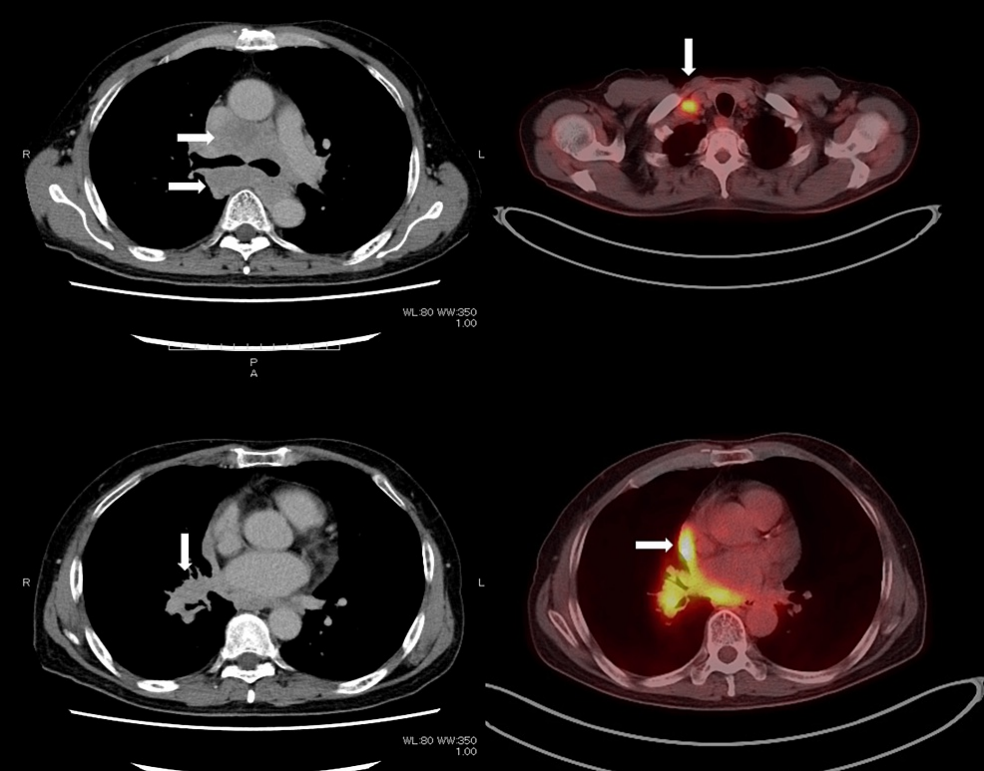
Imaging findings at the initial visit. Computed tomography (CT) scan at the initial visit showing a large mass consisting of mediastinal lymph node swelling (white arrows, top left) and a tumor in the right lower lobe (white arrow, bottom left). Positron emission tomography-CT also revealed positive lymph node metastasis in the right subclavian (white arrow, top right), as well as positive findings of cancerous pericarditis (white arrow, bottom right).

A 64-year-old man presented with a medical history of generalized anxiety and an inguinal hernia. He was referred from a community clinic with the chief complaint of cough. His social history included smoking for 60 pack years. Findings at the initial visit revealed widened tracheal bifurcation on chest X-ray and a large mass consisting of an approximately 4-cm tumor in the right lower lobe combined with bilateral hilar and mediastinal lymph nodes on computed tomography (CT). Right subclavian lymph node enlargement was also found, and positron emission tomography (PET-CT) demonstrated positive findings of lymph node metastasis in the corresponding areas indicated by the CT scans. PET-CT also revealed pericardial effusion and positive signals in the epicardium, suggestive of carcinomatous pericarditis (Figure 1).

**Figure 2:**
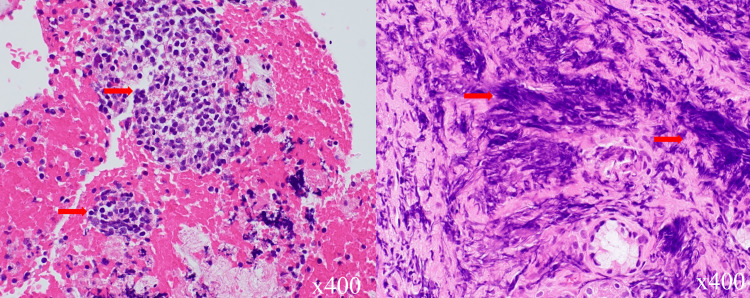
Histology findings of tissues obtained from diagnostic bronchoscopy. Transbronchial needle aspiration biopsy tissue showing atypical cells small and uniform in size with a high nuclear-to-cytoplasmic ratio (red arrows, left). Transbronchial biopsy tissue also showing distorted and fragmented cells resulting from a crushed artifact (red arrows, right). Both findings demonstrate typical characteristics of small cell lung cancer (SCLC) histology.

Bronchoscopy demonstrated a dull bifurcation and narrowing of the airway in the right lower lobe, capillary dilation, and a tumor in the right lower lobe. Bronchial biopsy tissue from the right lower trunk, as well as ultrasound-guided transbronchial needle aspiration biopsy from the subcarinal lymph node, revealed cells rich in chromatin relatively in a smaller size, as well as spindle-shaped nuclei, which is compatible with the histological diagnosis of small cell carcinoma (Figure 2).

Diagnostic immunohistochemistry revealed strong positivity for CD56NCAM and negativity for synaptophysin and chromogranin, suggesting that the cancer tissue does not demonstrate phenotypes of large-cell neuroendocrine differentiation. Immunohistochemistry was negative for PD-L1 protein with TPS and taken together with the spread of the disease, the patient’s condition was determined to be ES-SCLC with clinical TNM stage T4N3M1a. Laboratory data at the initial visit are shown in Table 1.

**Table 1:** Peripheral blood findings at the time of admission. ProGRP: progastrin-releasing peptide, CEA: carcinoembryonic antigen, CA19-9: cancer antigen 19-9

Peripheral blood findings	
White blood cells	4800/ul
Red blood cells	4.77×10^6^/ul
Hemoglobin	15.5 g/dL
Hematocrit	46.0%
Platelet	216×10^3^/ul
Mean corpuscular volume	96 fl
Mean corpuscular hemoglobin	32.5 pg
Mean corpuscular hemoglobin concentration	33.7%
Red blood cell distribution width	13.8%
Mean platelet volume	9.5 fl
Basophils	0.8%
Eosinophils	4%
Neutrophils	75.5%
Lymphocytes	12.6%
Monocytes	7.1%
Aspartate aminotransferase	20 U/L
Alanine transaminase	20 U/L
Lactate dehydrogenase	173 U/L
Glucose	83 mg/dL
Blood urea nitrogen	16 mg/dL
Creatinine	0.93 mg/dL
Sodium	142 mEq/L
Potassium	4.8 mEq/L
Chloride	104 mEq/L
C-reactive protein	0.03 mg/dL
ProGRP	59.5 pg/mL
CEA	1.7 ng/mL
CA19-9	11.4 U/mL

**Figure 3:**
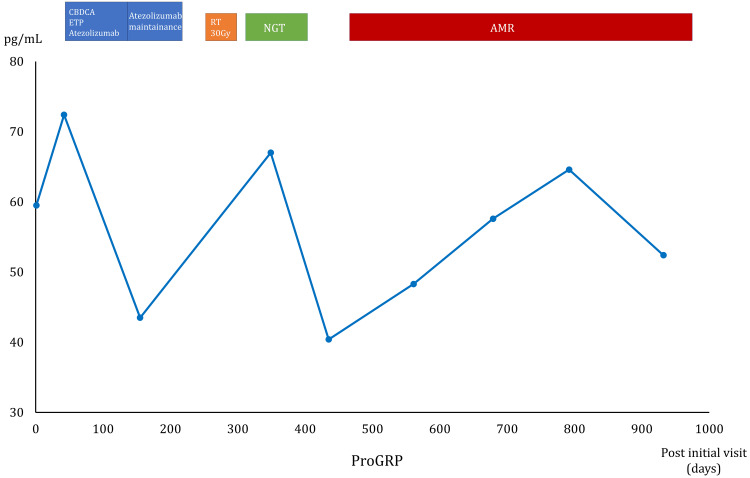
The clinical course of the patient's treatment. The clinical course of the patient’s treatment of extensive-stage small cell lung cancer (ES-SCLC). The patient received his first-line treatment with carboplatin, etoposide, and atezolizumab for four cycles, followed by atezolizumab administration every three weeks (blue rectangles). Following the worsening of his condition with pleural effusion, the patient received palliative irradiation (orange rectangle) followed by nogitecan for four cycles (green rectangle). The patient experienced a second cancer recurrence treated with amrubicin (red rectangle). His tumor marker ProGRP levels remain stable throughout the clinical course as indicated by the line graph. ProGRP: progastrin-releasing peptide

**Figure 4:**
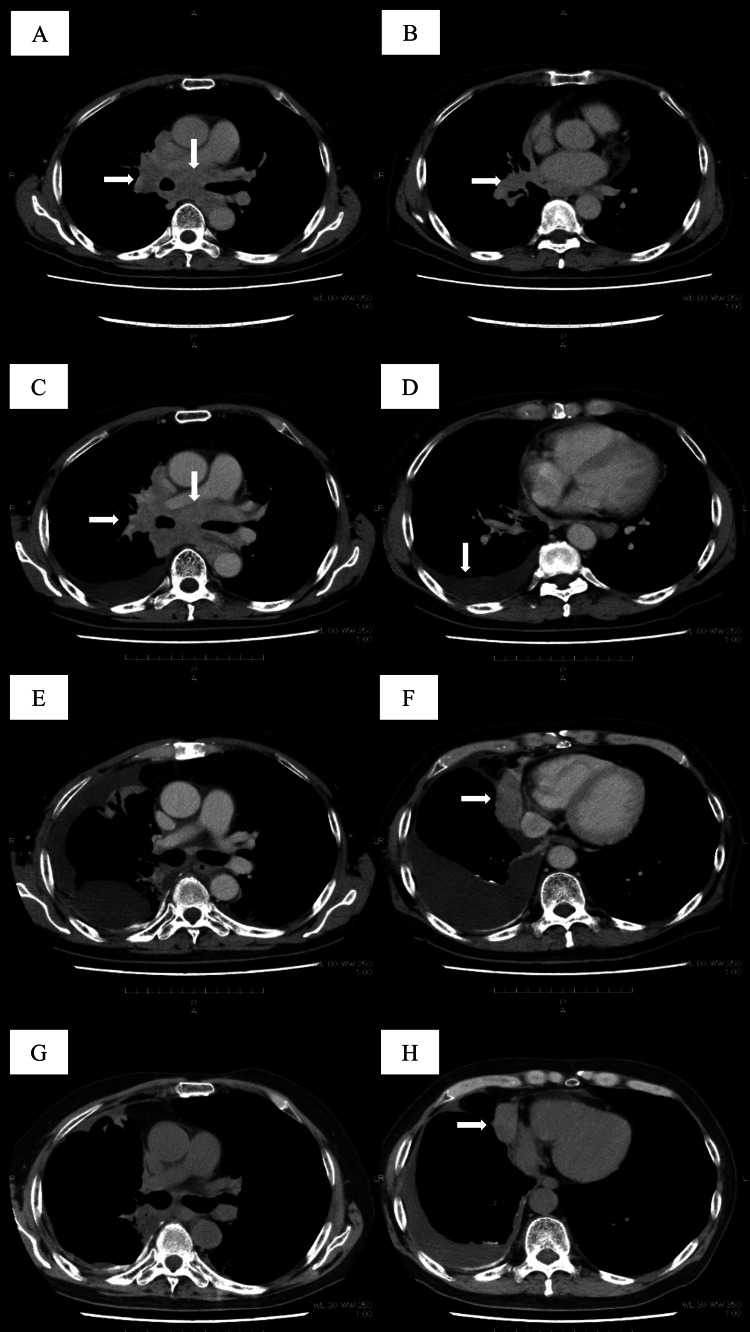
CT scan findings during the patient's treatment. Areas of interest described are demonstrated by the white arrows. (A)(B) Computed tomography (CT) scan findings before the first line of chemotherapy demonstrate findings of mediastinal lymph node enlargement and a right lower lobe tumor. (C)(D) Six months after initiation of his first-line chemotherapy, a newly diagnosed pleural effusion was revealed, while his mediastinal lymph nodes and right lower lobe tumor remained stable. (E)(F) Prior to the administration of his third-line amrubicin treatment, his previous therapy with palliative irradiation, four cycles of nogitecan, and pleurodesis result in fair control of the right pleural effusion. However, a newly found tumor in his right lung adjacent to the heart is revealed. (G)(H) Imaging studies reveal stable findings 16 months following the initiation of his third-line treatment with amrubicin.

The time-lapse of the patient’s clinical course and pertinent imaging findings are summarized in Figure 3 and Figure 4.

The patient’s treatment for lung cancer was initiated with carboplatin (470mg day 1), etoposide (130mg days 1-3), and atezolizumab (1200mg day 1) for four cycles monthly, followed by the administration of atezolizumab (1200mg day 1) every three weeks. The general condition of the patient, as well as the pulmonary and mediastinal lesions, remained grossly unchanged without progression, and his treatment outcome was evaluated as a stable disease during first-line chemotherapy.

Six months after initiation of treatment, pleural effusion became relevant; thoracentesis demonstrated chylous effusion, and laboratory studies of his pleural fluid were identified as class II with no malignant cells. Biochemical analysis of his pleural effusion included cell counts of 2559/uL with a negative Rivalta test and lymphocyte percentage of 92%, triglyceride levels of 117 mg/dL, and hyaluronic acid levels of 26945 ng/mL (Table 2).

**Table 2:** Chest fluid findings at the time of thoracentesis. CEA: carcinoembryonic antigen, pH: power of hydrogen

Chest fluid findings	
Adenosine deaminase	21.8 U/L
Hyaluronic acid	26945 ng/mL
Protein	3.7 g/dL
Glucose	91 mg/dL
Rivalta	(-)
Chest fluid density	1.016
Lactate dehydrogenase	148 U/L
Amylase	39 U/L
Triglyceride	117 mg/dL
Sodium	147 mEq/L
Potassium	3.7 mEq/L
Chloride	114 mEq/L
CEA	2.4 ng/mL
Cell count	2559/µL
Neutrophils	3%
Lymphocytes	92%
Eosinophils	0%
Monocytes	0%
pH	8.5

We diagnosed his newly developed chylothorax as a clinical recurrence, and palliative radiotherapy was performed with 30 Gy/10Fr irradiation to the mediastinal lymph nodes and right hilar tumor. Pleurodesis of the right thorax was also performed, and improvement in his pleural effusion was observed. The patient subsequently received second-line chemotherapy with monthly administration of nogitecan (1.6mg days 1-5) repeated for four cycles, while we observed improvement in his mediastinal lymph node findings. The condition of the right pleural effusion also remained stable following pleurodesis.

**Figure 5:**
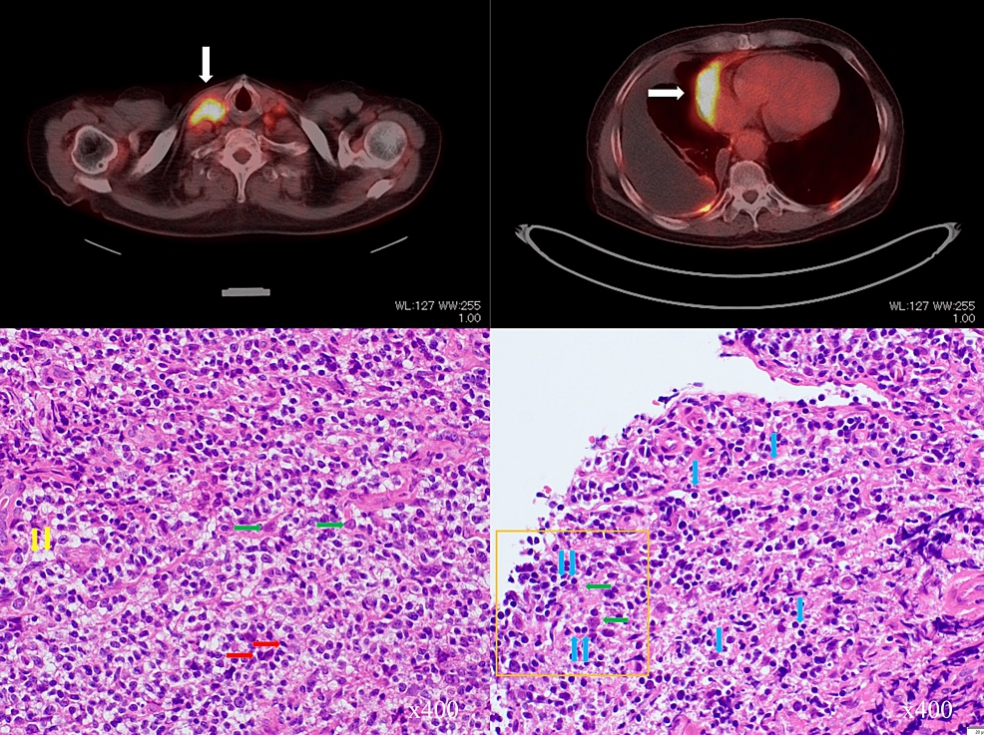
Positron emission tomography-CT and histological findings during the patient's third-line treatment. Positron emission tomography-CT during the patient's third-line treatment with amrubicin demonstrates high signal intensity in the right subclavian lymph node which has been persistent since the time of diagnosis (white arrow, top left), as well as positive findings in the tumor at his right lung adjacent to the heart (white arrow, top right) that was revealed upon initiating treatment with amrubicin. Histology of the patient's supraclavicular lymph node biopsy during his third-line treatment with amrubicin reveals viable cancer cells (red arrows, lower left); however, two-thirds of the cells in view were undergoing necrotic changes or cellular degeneration (green arrows, lower left). Vacuolar changes are also found in the tissue (yellow arrows, lower left), representing a therapeutic effect equivalent to Ef.2 (moderate) in the biopsy tissue. In visual fields where normal morphology lymph nodes are dominant (blue arrows, lower right), cancer cells undergoing degeneration (green arrows, lower right) surrounded by immune cell infiltration are also found (orange rectangle). CT: computed tomography

Two months after the final treatment with nogitecan, tumor regrowth was observed in the right lower lobe, leading to a diagnosis of clinical recurrence. He was started on third-line chemotherapy with amrubicin (65mg days 1-3) every four weeks. PET-CT performed 14 months after the second recurrence demonstrated remarkable findings of cervical and supraclavicular lymph node metastasis that persisted since the patient's initial visit, as well as positive findings in the right lower lobe mass which is the evaluation lesion during treatment with amrubicin. However, the size of the right lower lobe legion remained relatively stable since it appeared. Percutaneous biopsy of the right supraclavicular lymph node was obtained 14 months after initiation of amrubicin treatment, and the histological findings at this time demonstrated infiltration of cells rich in chromatin and spindle cell-shaped malignant cells, although approximately 2/3 of the cells in vision were under necrotic change or cellular degeneration, which is consistent with a moderate therapeutic effect (Figure 5).

The patient continued to receive treatment with amrubicin for a total of 20 months which is an extreme outlier considering that ES-SCLC recurrence treated with amrubicin demonstrates a nine-month progression-free survival rate of 10% and median overall survival of 7.5 months [8,9]. He remains in fair condition as of today, 35 months after his initial diagnosis.

## Discussion

We treated a case of ES-SCLC with an outstanding survival outcome of nearly three years, despite experiencing multiple worsening of his status. The administration of ICI as a first-line therapy lasted for a total of seven months. This was followed by the development of chylothorax and tumor regrowth, which prompted us to withdraw from immunotherapy and switch to other treatment options, including pleurodesis, palliative irradiation, and cytotoxic chemotherapy. He did not experience known adverse effects from atezolizumab such as endocrine disorders or systemic autoimmune response during his treatment.

His clinical course is worth noting, considering that ES-SCLC patients typically have a poor prognosis with a median survival of 10 months and a five-year survival rate of less than 2% [10,11]. Classical features proposed as favorable outcome characteristics in SCLC include disease in earlier stages and a good response to initial therapy [12]. Histology may also determine prognosis, and combined SCLC findings may result in a less favorable outcome [13]. In our case, other than histopathological findings not demonstrating combined SCLC or neuroendocrine differentiation, the patient’s clinical staging of ES-SCLC and response to initial therapy, not resulting in size reduction of the tumor, would not indicate a better prognosis. The fact that the patient experienced an exceptional survival outcome suggests that previously overlooked factors may have been involved.

The prior use of atezolizumab, an ICI, is a notable feature. The addition of ICI to chemotherapy in the first-line treatment of ES-SCLC results in significantly longer overall survival and progression-free survival compared to conventional chemotherapy [14]. While continued use of ICI may improve survival in patients with ES-SCLC, the prognosis of patients who drop out of the ICI treatment regimen remains unclear. One remarkable finding is that these studies combining PD-1/PD-L1 inhibitors with first-line chemotherapy demonstrated improvement in survival, while treatment with ICI as a post-second-line regimen did not demonstrate favorable outcomes [15].

Delayed therapeutic effects are often observed in patients undergoing ICI therapy. One hypothesis to understand this phenomenon is that the anticancer effects of checkpoint inhibitors can continue even after treatment is discontinued through reprogramming of the immune system by ICI [16], allowing it to better recognize cancer cells and demonstrate latent treatment effects. Some patients also experience a temporary increase in the size of the metastases despite experiencing overall improvement, a phenomenon known as pseudo-progression [17]. Therefore, standard evaluation criteria applying radiologic findings (RECIST-1.1) may not be applicable in patients undergoing ICI treatment, although new guidelines known as iRECIST (immune RECIST) [18], including an extended delay to evaluate therapeutic response, have been established. Our patient also experienced an outstanding outcome despite his initial biopsy being negative for PD-L1, which is the target molecule for atezolizumab, an ICI administered during the initial chemotherapy. It is possible that earlier modulation of the cancer immune environment can contribute to the treatment outcome in ES-SCLC patients; therefore, first-line treatment, including ICI, can improve survival even after discontinuation.

One major limitation of our study is that the methods to assess changes in the immune status prior to and after the administration of ICI are not well established, and there may have been other factors that may have contributed to the exceptional survival outcome of this patient. While prospective studies are needed to prove the superiority of ICI combined therapy as a first-line treatment for ES-SCLC after discontinuation, it should be stressed that histology samples obtained two years after the initiation of first-line therapy, including ICI, continued to demonstrate the effects of antitumor treatment. With the advancement of therapy for ES-SCLC patients, regimens, including ICI, may offer superiority for a wider population even years after the discontinuation of treatment.

## Conclusions

Here, we report a case of ES-SCLC with excellent outcomes. Upon admission, his prognosis was expected to be poor; however, he survived for over 30 months and continued to receive anticancer treatment. ICIs, among other therapies administered, may demonstrate delayed antitumor effects and significantly improve survival months after discontinuation, and the administration of ICIs as first-line therapy may result in favorable outcomes even in patients who experience multiple recurrences.
